# Fisetin Protects C2C12 Mouse Myoblasts from Oxidative Stress-Induced Cytotoxicity through Regulation of the Nrf2/HO-1 Signaling

**DOI:** 10.4014/jmb.2212.12042

**Published:** 2023-01-20

**Authors:** Cheol Park, Hee-Jae Cha, Da Hye Kim, Chan-Young Kwon, Shin-Hyung Park, Su Hyun Hong, EunJin Bang, Jaehun Cheong, Gi-Young Kim, Yung Hyun Choi

**Affiliations:** 1 Division of Basic Sciences, College of Liberal Studies, Dong-eui University, Busan 47340, Republic of Korea; 2 Department of Parasitology and Genetics, Kosin University College of Medicine, Busan 49267, Republic of Korea; 3 Anti-Aging Research Center and Core-Facility Center for Tissue Regeneration, Dong-eui University, Busan 47340, Republic of Korea; 4 Department of Molecular Biology, Pusan National University, Busan 46241, Republic of Korea; 5 Department of Oriental Neuropsychiatry, Dong-eui University College of Korean Medicine, Busan 47227, Republic of Korea; 6 Department of Pathology, Dong-eui University College of Korean Medicine, Busan 47227, Republic of Korea; 7 Department of Biochemistry, College of Korean Medicine, Dong-eui University, Busan 47227, Republic of Korea; 8 Department of Marine Life Science, Jeju National University, Jeju 63243, Republic of Korea

**Keywords:** Fisetin, oxidative stress, DNA damage, apoptosis, heme oxygenase-1

## Abstract

Fisetin is a bioactive flavonol molecule and has been shown to have antioxidant potential, but its efficacy has not been fully validated. The aim of the present study was to investigate the protective efficacy of fisetin on C2C12 murine myoblastjdusts under hydrogen peroxide (H_2_O_2_)-induced oxidative damage. The results revealed that fisetin significantly weakened H_2_O_2_-induced cell viability inhibition and DNA damage while blocking reactive oxygen species (ROS) generation. Fisetin also significantly alleviated cell cycle arrest by H_2_O_2_ treatment through by reversing the upregulation of p21WAF1/CIP1 expression and the downregulation of cyclin A and B levels. In addition, fisetin significantly blocked apoptosis induced by H_2_O_2_ through increasing the Bcl-2/Bax ratio and attenuating mitochondrial damage, which was accompanied by inactivation of caspase-3 and suppression of poly(ADP-ribose) polymerase cleavage. Furthermore, fisetin-induced nuclear translocation and phosphorylation of Nrf2 were related to the increased expression and activation of heme oxygenase-1 (HO-1) in H_2_O_2_-stimulated C2C12 myoblasts. However, the protective efficacy of fisetin on H_2_O_2_-mediated cytotoxicity, including cell cycle arrest, apoptosis and mitochondrial dysfunction, were greatly offset when HO-1 activity was artificially inhibited. Therefore, our results indicate that fisetin as an Nrf2 activator effectively abrogated oxidative stress-mediated damage in C2C12 myoblasts.

## Introduction

Reactive oxygen species (ROS) are generally maintained at appropriate levels in the cells and act as regulators of signaling pathways involved in maintaining cellular homeostasis. However, excessive accumulation of ROS, defined as oxidative stress, causes irreversible oxidative damage to proteins, lipids, DNA and organelles, ultimately leading to apoptosis [1, 2]. Therefore, it is necessary to strictly control the level of ROS through antioxidants or intracellular antioxidant systems to maintain normal cell function [3, 4]. Recently, polyphenols, which are abundant in the plant kingdom, have been found to have various pharmacological effects, including inhibition of oxidative stress-dependent pathological conditions. Their antioxidant activity mainly involves ROS scavenging and activation of intracellular antioxidant signaling pathways, and can terminate oxidative damage-mediated responses before cells are severely affected for survival [5, 6].

Among polyphenols, fisetin, a flavonol molecule, has shown health-improving potential in numerous previous studies [7, 8]. This phytochemical is found as a coloring component in fruits and vegetables such as apples, cucumbers, onions, and persimmons. According to accumulated studies, fisetin is known to have multiple pharmacological effects, including neuroprotective, antidiabetics, antiallergic, antithrombotic, senotherapeutic, anti-inflammatory, cardioprotective, neurodegenerative, cancer chemopreventive and ant-cancer activities [8-10]. The involvement of numerous intracellular molecules and signaling systems in the antioxidant efficacy of fisetin has also been confirmed in several experimental models. Among them, the idea that activation of heme oxygenase-1 (HO-1), an antioxidant enzyme controlled by nuclear factor-erythroid-2 related factor 2 (Nrf2), might play a key action is based on the report of Hanneken *et al*. [11]. According to their results, fisetin significantly blocked hydrogen peroxide (H_2_O_2_)-mediated cell death in human retinal pigment epithelial (RPE) cells associated with the increased HO-1 and Nrf2 expression. Based on their findings, we suggested that that activation of the Nrf2/HO-1 axis by fisetin in H_2_O_2_-treated RPE cells was directly associated with ROS scavenging activity [12]. Recently, it has been shown that the reduction of ROS generation by fisetin could contribute to the alleviation of neointimal hyperplasia after intimal injury by inhibiting proliferation and invasion of smooth muscle cells in blood vessels [13]. Additionally, Rodius *et al*. [14] reported that fisetin inhibited apoptosis and DNA damage in cardiomyocytes subjected to hypoxia/starvation-reoxygenation while reducing oxidative stress. Moreover, it has been demonstrated that ROS scavenging activity of fisetin, which is related to the increased transcriptional activity of HO-1, was responsible for the alleviation of phenylephrine-induced cardiomyopathy [15]. Nevertheless, the antioxidant and protective roles of fisetin on muscle cells have not been elucidated in detail. Therefore, in this study, we evaluated the cytoprotective effect of fisetin in C2C12 murine myoblasts treated with H_2_O_2_ to mimic oxidative stress.

## Materials and Methods

### Cell Culture and Cell Viability

C2C12 myoblasts (CRL-1772, ATCC, USA) were cultured as previously described [16]. To analyze the protective effect of fisetin (Sigma-Aldrich Co., USA) on oxidative stress caused by H_2_O_2_ (Sigma-Aldrich Co.), C2C12 cells were treated with fisetin and H_2_O_2_ for 24 h, or cells pretreated with fisetin, *N*-acetyl-L-cysteine (NAC, Thermo Fisher Scientific, USA) or zinc protoporphyrin IX (ZnPP, Sigma-Aldrich Co.) for 1 h were further treated with H_2_O_2_ for 24 h. To evaluate the inhibitory action of fisetin on ROS accumulation by H_2_O_2_, cells were treated with fisetin or NAC for 1 h and then treated with H_2_O_2_ for 1 h. 3-(4,5-dimethylthiazol-2-yl)-2,5-diphenyltetrazolium bromide (MTT, Thermo Fisher Scientific) assay was used to investigate the protective potential of fisetin on the suppression of cell viability by H_2_O_2_ according to the previously described method [17]. Morphological changes of cells were observed using a phase-contrast microscope (Olympus, Japan). Stock solutions of fisetin and H_2_O_2_ were prepared by dissolving in dimethyl sulfoxide (DMSO, Sigma-Aldrich Co.), and the highest concentration of DMSO was less than 0.05%, showing no cytotoxicity.

### ROS Generation Assay

For quantitative evaluation of ROS generation, cells were stained with 10 μM 2’,7’-dichlorofluorescein diacetate (DCF-DA, Thermo Fisher Scientific) and then the intensity of DCF fluorescence reflecting ROS production was analyzed as described previously [18]. In addition, DCF-DA fluorescence images were captured under a fluorescent microscope (Carl Zeiss, Germany).

### Comet Assay

To examine the suppressive effect of fisetin on DNA damage induced by H_2_O_2_, Comet Assay Kit (Trevigen, Inc., USA) was used following the manufacturer’s protocol. We also used OpenComet software v1.3.1 [https://cometbio.org/] to analyze comet images.

### 8-Hydroxy-2’-Deoxyguanosine (8-OHdG) Assay

In order to measure the amount of 8-OHdG used as a representative marker for oxidative DNA damage, an OxiSelect Oxidative DNA Damage Kit (Cell Biolabs, USA) was used. Briefly, according to the protocol presented in the kit, DNA extracted from cells was digested with DNase I and absorbance was calculated at 450 nm.

### Immunoblotting Assay

Total protein for analysis of protein expression by immunoblotting was extracted according to the previous method [19]. Mitochondrial Fractionation Kit and NE-PER Nuclear and Cytoplasmic Extraction Kit purchased from Thermo Fisher Scientific and Sigma-Aldrich Co., respectively, were used for separation of cytoplasmic, mitochondrial and nuclear fractions. Immunoblotting using the isolated proteins was performed according to the same method as described previously [19]. Primary antibodies against Nrf2 (Mouse monoclonal, 1:1000, sc-518036), HO-1 (Mouse monoclonal, 1:1000, sc-136960), p21 (Mouse monoclonal, 1:1000, sc-271610), cyclin A (Mouse monoclonal, 1:1000, sc-271645), Bax (Mouse monoclonal, 1:1000, sc-7480), Bcl-2 (Mouse monoclonal, 1:1000, sc-509), caspase-3 (Mouse monoclonal, 1:1000, sc-56052), poly(ADP-ribose) polymerase (PARP, mouse monoclonal, 1:1000, sc-8007), cytochrome c (Mouse monoclonal, 1:500, sc-13560), actin (Mouse monoclonal, 1:1000, sc-47778) and secondary antibodies were from Santa Cruz Biotechnology Inc. (USA). Anti-cyclin B1 (Rabbit polyclonal, 1:1000, #4138, cyclin-dependent kinase (Cdk) 2 (Rabbit polyclonal, 1:1000, #2546), Cdc2 (Rabbit polyclonal, 1:1000, #9112) and cytochrome oxidase subunit 4 (COX IV, rabbit polyclonal, 1:1000, #4844) antibodies were purchased from Cell Signaling Technology Inc. (USA). Primary antibodies against histone deacetylase 2 (HDAC2, rabbit monoclonal, 1:1000, ab32117) and heat shock protein 90 (HSP90, mouse monoclonal, 1:1000, ab13492) were Abcam, Inc. (UK). Phospho (p)-Nrf2 (Rabbit polyclonal, 1:500, PA5-67520) antibody was obtained from Thermo Fisher Scientific Inc.

### Immunofluorescence Assay

For immunofluorescent staining of γH2AX (Ser139) to observe DNA damage, the collected cells were reacted with anti-γH2AX antibody (Rabbit polyclonal, #11585573, Thermo Fisher Scientific) and Alexa Fluor 594-conjugated antibody (Rabbit polyclonal, #8889, Cell Signaling Technology Inc.). After counterstaining the nuclei using 6-diamidino-2-phenylindol (DAPI, Thermo Fisher Scientific Inc.), images were acquired [18].

### HO-1 Activity Assay

To investigate the activity of HO-1, HO-1 ELISA Kit (Abcam Inc.) was used. Briefly, the amount of bilirubin formed in the heme was evaluated using cell lysates, and the activity of HO-1 was expressed as fold change relative to the untreated control.

### Flow Cytometry Analysis

To investigate cell cycle distribution of cells cultured under different conditions, the fixed-cells were stained with propidium iodide (PI, Thermo Fisher Scientific) [20]. After staining, the frequency of cells corresponding to each cell cycle phase was calculated using a flow cytometer (BD Biosciences, USA). For quantitative evaluation of cellular apoptosis, Annexin V-FITC/PI Apoptosis Assay Kit (Abcam Inc.) was used. According to the method presented in the kit, the collected cells were reacted with annexin-V/PI solutions. Then, annexin V+ cells were regarded as cells in which apoptosis was induced [20]. To detect changes in mitochondrial membrane potential (MMP), 5,5’,6,6’-tetrachloro-1,1’3,3’-tetraethyl-imidacarbocyanune iodide (JC-1, Sigma-Aldrich Co.) was used. After flow cytometry analysis, the loss of MMP was indicated as a percentage of JC-1 monomers.

### Statistical Analysis

All statistical analyses were performed using GraphPad Prism 8.0 (GraphPad, USA) according to the manufacturer's instructions. Data are expressed as mean ± standard deviation (SD) and *p* < 0.05 was considered a statistically significant difference.

## Results

### Restoration of H_2_O_2_-Induced Loss of Cell Viability by Fisetin

**Fig. 1:**
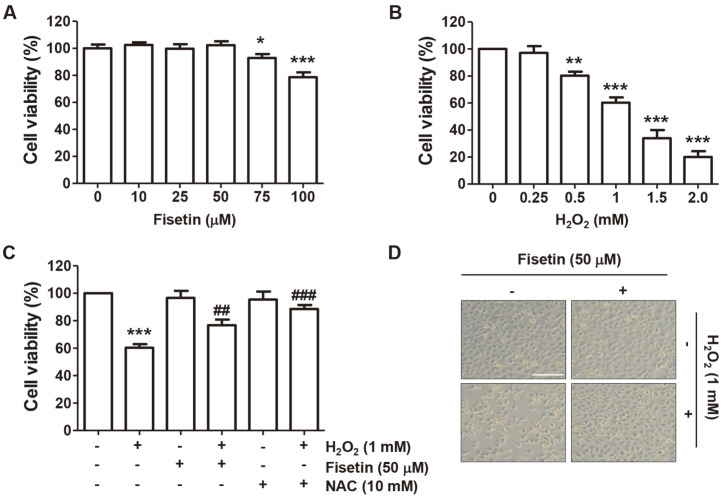
Fisetin inhibited the reduction of cell viability caused by H_2_O_2_ treatment in C2C12 cells. (**A-C**) MTT assay was performed after cells were treated with various concentrations of fisetin and H_2_O_2_ for 24 h (**A** and **B**) or pretreated with or without fisetin and NAC for 1 h followed by stimulation with H_2_O_2_ for an additional 24 h (**C**). *p < 0.05, **p < 0.01 and ***p < 0.001 *vs.* control group; ^##^*p* < 0.01 and ^###^*p* < 0.001 *vs.* H_2_O_2_-treated cells. (**D**) Representative morphological images of cells exposed to H_2_O_2_ in the presence or absence of fisetin were presented (200x). Scale bar is 50 μm.

To investigate the inhibitory effect of fisetin on H_2_O_2_-mediated oxidative stress in C2C12 cells, we evaluated the effects of fisetin and H_2_O_2_ on cell viability. As shown in Fig. 1A, fisetin did not induce significant suppression of cell survival at a concentration of up to 50 μM, but cytotoxicity was observed at concentrations higher than that (Fig. 1A), so the optimal concentration for evaluating the inhibitory efficacy against H_2_O_2_-induced cytotoxicity was set to 50 μM. The dose of H_2_O_2_ treatment for inducing oxidative damage was set at 1 mM, which showed a cell viability of approximately 60% compared to untreated control (Fig. 1B). Next, we evaluated the protective effect of fisetin on inhibition of cell viability induced by H_2_O_2_, and found that fisetin pretreatment significantly restored the reduced cell survival in cells treated with H_2_O_2_. In addition, H_2_O_2_-mediated cytotoxicity was completely inhibited in the presence of NAC, a free radical scavenger (Fig. 1C). Furthermore, cells treated with H_2_O_2_ became elongated and lost adhesion, and the number of cells floated on the medium increased, but not in cells exposed to H_2_O_2_ after fisetin pretreatment (Fig. 1D).

### Suppression of H_2_O_2_-Induced ROS Production by Fisetin

**Fig. 2:**
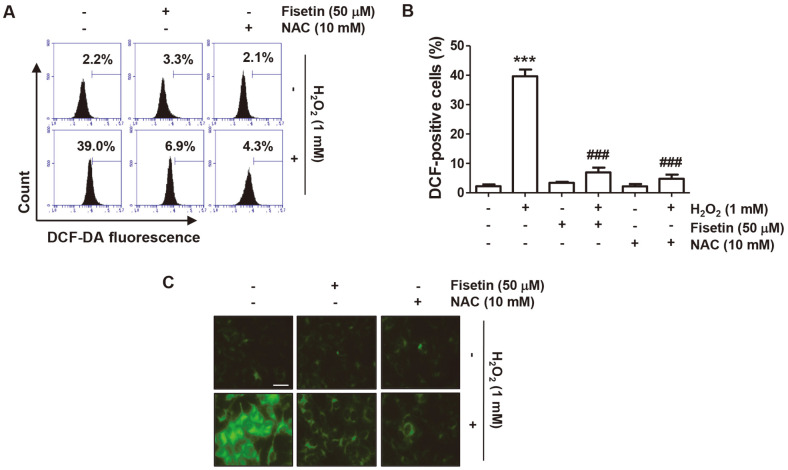
Fisetin attenuated ROS production in H_2_O_2_-treated C2C12 cells. Cells exposed with or without fisetin and NAC for 1 h were stimulated with H_2_O_2_ for another 1 h. The level of ROS production was investigated by performing DCF-DA staining. (**A** and **B**) Representative flow cytometry histograms (**A**) and mean values of the data were presented (**B**). ***p < 0.001 *vs*. control group; ^###^*p* < 0.001 *vs.* H_2_O_2_-treated cells. (**C**) Representative immunofluorescence images following DCF-DA staining were indicated. Scale bar is 30 μm.

To evaluate whether the suppressed cell viability in H_2_O_2_-treated cells was related to the generation of ROS and whether fisetin can inhibit it, the level of intracellular peroxides was investigated with DCF-DA dye. Flow cytometry analysis indicated that the dramatically increased ROS production in H_2_O_2_-treated cells was markedly reduced in the presence of fisetin (Figs. 2A and 2B). Similar to this result, the DCF fluorescence intensity increased by H_2_O_2_ treatment was greatly decreased by fisetin pretreatment (Fig. 2C). In addition, the efficacy of fisetin to block ROS generation was similar to that of NAC used as a control.

### Blockade of H_2_O_2_-Induced DNA Damage by Fisetin

**Fig. 3:**
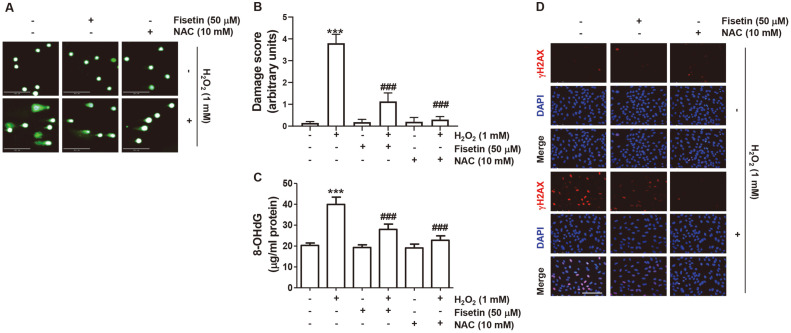
Fisetin alleviated DNA damage in H_2_O_2_-treated C2C12 cells. Cells exposed with or without fisetin and NAC for 1 h were stimulated with H_2_O_2_ for another 24 h. (**A**) Representative immunofluorescence images following comet assay were indicated. Scale bar is 250 μm. (**B**) Result of DNA damage score using OpenComet software. Data indicate mean ± SD values (*n* = 3; ***p < 0.001 *vs.* control cells; ^###^*p* < 0.001 *vs.* H_2_O_2_‐treated cells). (**C**) After treatment, contents of 8- OHdG, which is the deoxyriboside form of 8-oxoGuanine, were measured. (**D**) After performing fluorescence staining to evaluate the expression of γH2AX (red), the nuclei were further stained with DAPI (blue) and visualized with a fluorescence microscope. Scale bar is 100 μm.

We subsequently determined whether the effect of fisetin to block H_2_O_2_-induced generation of ROS was associated with blocking DNA damage. As indicated in Fig. 3, in H_2_O_2_-treated cells, an increase in comet tail moment (Figs. 3A and 3B), 8-OHdG content (Fig. 3C) and γH2AX (Ser139) expression (Fig. 3D) were clearly observed. However, the increase in these DNA damage markers by H_2_O_2_ was effectively counteracted by pretreatment with NAC as well as fisetin.

### Activation of the Nrf2/HO-1 Antioxidant Signaling by Fisetin

**Fig. 4:**
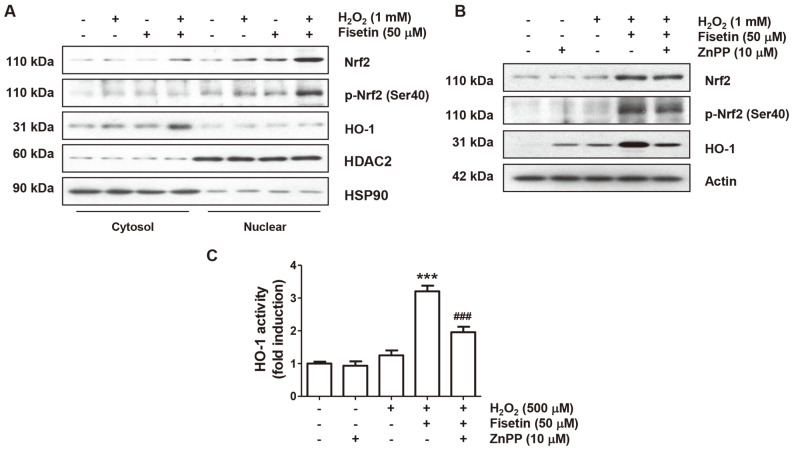
Fisetin activated Nrf2/HO-1 signaling pathway in H_2_O_2_-treated C2C12 cells. Cells were incubated for 1 h in medium with or without fisetin or ZnPP and then treated with H_2_O_2_ for 24 h. After extracting the cytoplasmic and nuclear proteins (**A**) or total proteins (**B**) for each treatment group, the expression levels of the presented proteins were investigated by immunoblotting. (**C**) HO-1 activity was presented as a relative value. ***p < 0.001 *vs.* control group; ^###^*p* < 0.001 *vs.* fisetin + H_2_O_2_ treatment group.

Next, we investigated whether the Nrf2/HO-1 signaling was involved in the antioxidant capacity of fisetin. As shown in Fig. 4A, the levels of Nrf2 and its phosphorylated form (p-Nrf2, Ser40) were slightly upregulated in the nuclei of cells stimulated with either fisetin or H_2_O_2_ alone. However, in cells treated with H_2_O_2_ and fisetin, they were remarkedly upregulated compared to cells treated with each alone. Moreover, the level of HO-1 protein was clearly enhanced in the cytoplasm of H_2_O_2_-treated cells after pretreatment with fisetin, and the activity of HO-1 was also promoted (Fig. 4C), which were restored again in cells pretreated with ZnPP, an HO-1 inhibitor, and fisetin (Figs. 4B and 4C).

### Restoration of H_2_O_2_-Induced Cell Cycle Arrest and Apoptotic Cell Death by Fisetin

**Fig. 5:**
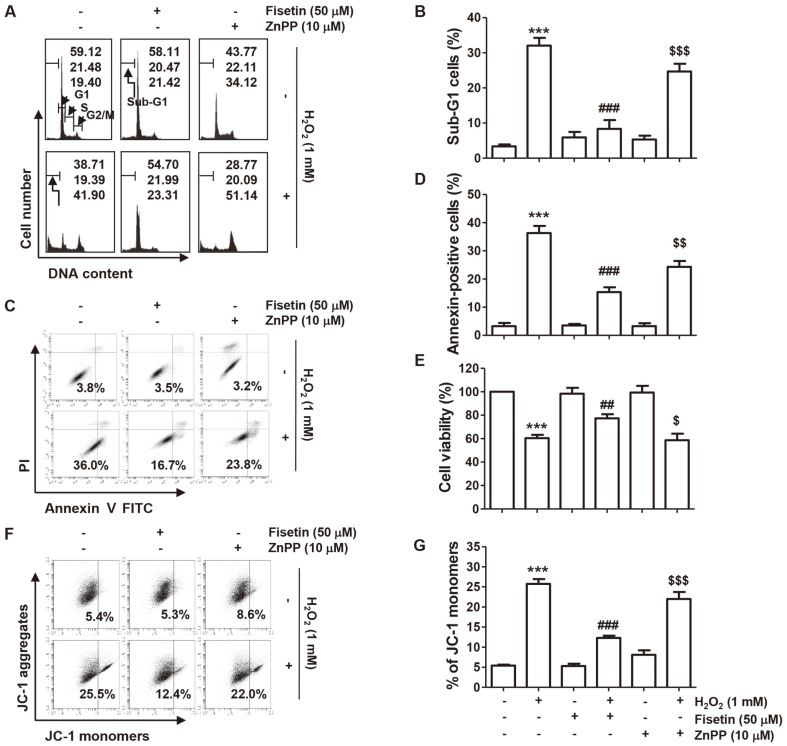
Fisetin ameliorated H_2_O_2_-induced cell cycle arrest, apoptosis and mitochondria impairment in C2C12 cells. Cells were cultured for 1 hour in medium containing fisetin and ZnPP or not, and then treated with H_2_O_2_ for an additional 24 h. Cell cycle distribution, induction of apoptosis and changes in MMP were evaluated by flow cytometry. (**A** and **B**) The frequencies of cells belonging to each stage of the cell cycle (**A**) and the sub-G1 phase, which is the apoptosis index, were shown (**B**). (**C** and **D**) After staining with annexin V/PI, flow cytometry was performed, and representative histograms (**C**) and the results of quantitative analysis (**D**) were shown. (**E**) Cell viability of cells cultured under the same conditions was assessed by the MTT assay. (**F** and **G**) After JC-1 staining, representative flow cytometry histograms were indicated (**F**), and the ratio of JC- 1 monomers in cells in each treatment group was expressed as mean ± SD (**G**). ***p < 0.001 *vs.* control group; ^##^*p* < 0.01 and ^###^*p* < 0.001 *vs.* H_2_O_2_-treated cells; ^$^*p* < 0.05, ^$$^*p* < 0.01 and ^$$$^*p* < 0.001 *vs.* fisetin + H_2_O_2_ treatment group.

We further examined the efficiency of fisetin on H_2_O_2_-induced of cell cycle arrest and apoptosis. As demonstrated in Figs. 5A and 5B, the frequencies of cells distributed in the G2/M and sub-G1 phases were significantly increased by H_2_O_2_ treatment, whereas the frequencies of the G1 and S phases were relatively decreased. In parallel, from the flow cytometry results, it was confirmed that apoptosis induction was significantly increased in H_2_O_2_-exposed cells than in control cells (Figs. 5C and 5D). However, cell cycle arrest and apoptosis induced by H_2_O_2_ were markedly reduced in cells in the presence of fisetin, and these blocking effects of fisetin were neutralized by ZnPP (Figs. 5A-5D). The inhibitory potential of fisetin on cell viability inhibition by H_2_O_2_ was also attenuated by ZnPP (Fig. 5E).

### Inhibition of H_2_O_2_-Induced Expression Changes of Regulators of Cell Cycle and Apoptosis by Fisetin

**Fig. 6:**
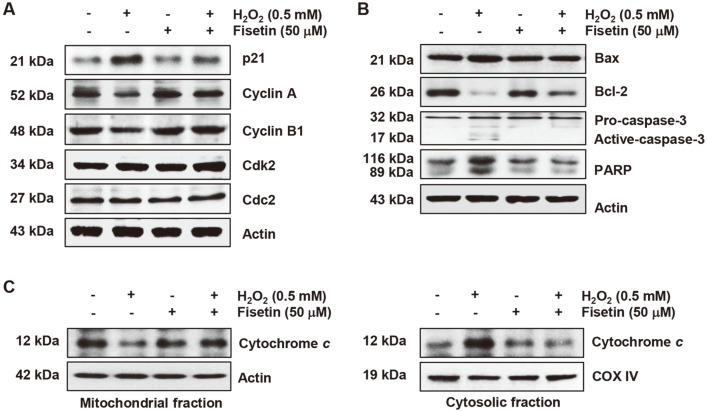
Fisetin counteracted changes in the expression of key regulators of cell cycle and apoptosis in H_2_O_2_- treated C2C12 cells. Cells were pretreated with or without fisetin for 1 h prior to treatment with H_2_O_2_ for 24 h. After extracting the total protein (**A** and **B**) or mitochondrial and cytoplasmic proteins (**C**) from each treatment group, the expression levels of the presented proteins were investigated by immunoblotting.

We also investigated the inhibitory effect of fisetin on changes in the expression of key regulators of cell cycle and apoptosis in C2C12 cells treated with H_2_O_2_. Immunoblotting results indicated that the level of p21WAF1/CIP1 protein, was upregulated by H_2_O_2_treatment, whereas the level of cyclin A and cyclin B1 proteins was downregulated without changes in the level of Cdc2 (cyclin-dependent kinase 1, Cdk1) and Cdk2 (Fig. 6A). Among the Bcl-2 family proteins, Bax expression was induced while Bcl-2 expression was inhibited (Fig. 6B), which was associated with activation of caspase-3 and degradation of PARP. However, these changes were offset in H_2_O_2_-treated cells after pretreatment with fisetin.

### Attenuation of H_2_O_2_-Induced Mitochondrial Impairment by Fisetin

Finally, we examined whether the protective efficacy of fisetin on H_2_O_2_-mediated cytotoxicity was related to the protection of mitochondrial dysfunction. JC-1 staining results showed that the level of JC-1 monomers was highly increased in H_2_O_2_-treated cells, suggesting that loss of MMP, indicating mitochondrial dysfunction, was caused (Figs. 5F and 5G). However, H_2_O_2_-induced loss of MMP was greatly alleviated by fisetin pretreatment, and this inhibitory effect was also abolished by ZnPP. Additionally, in H_2_O_2_-treated cells, cytochrome c expression was predominantly detected in the cytoplasm rather than mitochondria, and did not occur in cells pretreated with fisetin (Fig. 6C).

## Discussion 

Myoblasts, the embryonic precursors of skeletal muscle, differentiate into muscle cells through myogenesis that fuses into multinucleated myotubes [21, 22]. Although ROS can act as modulators of cellular signaling pathways required for muscle differentiation, excessive ROS production is strongly associated with impaired muscle formation. In addition, damage to myoblasts due to ROS accumulation contributes to blocking muscle differentiation and inducing muscle atrophy [23, 24]. Thus, the level of ROS must be regulated for the maintenance of muscle differentiation and function of myoblasts.

In this study, we induced oxidative stress using H_2_O_2_ to determine whether fisetin could protect C2C12 myoblasts from oxidative damage. Our results showed that fisetin, as an Nrf2 activator, blocks H_2_O_2_-induced cytotoxicity while scavenging ROS. Our results also demonstrated that H_2_O_2_-induced decrease in cell viability and ROS production in C2C12 cells were significantly alleviated by in the presence of fisetin or NAC, a scavenger of ROS used as a positive control. According to previous studies, DNA damage, cell cycle disruption and cell death are increased in myoblasts exposed to oxidative stress [24, 25]. Therefore, we first investigated whether fisetin could block H_2_O_2_-induced DNA damage by measuring DNA damage markers such as comet tail moment (DNA migration), p-γH2AX (Ser139) expression, and amount of 8-OHdG [26] and found that these three indicators were significantly increased in H_2_O_2_-treated C2C12 cells. However, all these changes were abrogated by fisetin pretreatment, and similar observations were made in NAC-pretreated cells. Our results well support those seen in H_2_O_2_-treated human retinal pigment epithelial cells and Chinese hamster lung fibroblasts, and hypoxia/starvation-exposed cardiomyocytes [12, 14, 27]. Therefore, our results showed that the ROS scavenging activity of fisetin may contribute to the inhibition of H_2_O_2_-induced DNA damage in C2C12 cells.

Nrf2 is a critical factor that controls the transcriptional activity of anti-oxidant enzymes involved in redox homeostasis [28, 29]. For nuclear translocation of Nrf2 to promote transcriptional activity of antioxidant response element (ARE)-mediated genes involved in defense against oxidative damage, Nrf2 must be phosphorylated [29, 30]. HO-1 is a representative downstream factor among detoxifying enzymes controlled by Nrf2 and can decompose toxic heme to biliverdin, carbon monoxide and free iron. The produced biliverdin is further converted to bilirubin, which has an antioxidant activity [28, 31]. These findings indicate that discovering substances that activate the Nrf2/HO-1 signaling may be one of the appropriate strategies to counteract oxidative stress-mediated cellular damage. Several previous studies have shown that fisetin was able to prevent DNA damage and apoptosis induced oxidative stress through regulation of Nrf2/HO-1 axis [12, 32, 33]. We therefore investigated whether fisetin could activate Nrf2 and found that the level of Nrf2 and p-Nrf2 (Ser40) was clearly upregulated in the nucleus of H_2_O_2_-treated C2C12 cells by fisetin. Concomitantly, the expression of HO-1 in the cytoplasm was enhanced and its activity was also significantly increased, indicating that fisetin acted as an Nrf2 activator that can promote the activity of HO-1.

As is well known, cytotoxicity by oxidative stimuli including H_2_O_2_ is accompanied by cell cycle arrest and apoptosis [34, 35]. Similar to our results, H_2_O_2_ treatment blocked cell cycle progression in the G2/M phase in most cell types, including C2C12 myoblasts. This is associated with increased expression of p21WAF1/CIP1, a Cdk inhibitor, and decreased expression of positive regulators required for G2 to M phase progression such as cyclin A and cyclin B1 [36, 37]. However, expression changes of these proteins and cell cycle arrest were significantly mitigated by fisetin pretreatment. In parallel with this, H_2_O_2_-induced apoptosis was due to activation of an intrinsic pathway mediated by mitochondrial impairment following the generation of ROS [38-40]. This pathway is activated by cytochrome c released from mitochondria into the cytosol following mitochondrial membrane depolarization, along with changes in the activity of Bcl-2 family proteins due to ROS overload [41, 42]. Cytochrome c sequentially activates caspases cascade, causing in cleavage of proteins including PARP, thereby terminating apoptosis [43, 44]. In this study, H_2_O_2_ treatment also induced a decrease in MMP and cytosolic release of cytochrome c, but not in the presence of fisetin. Moreover, the changes of Bax and Bcl-2 expression, and cleavage of PARP by H_2_O_2_ were maintained at control levels after fisetin pretreatment. These findings indicate that fisetin was able to prevent C2C12 myoblasts from cell cycle perturbation and cell death by blocking ROS generation due to oxidative stress. However, the blocking ability of fisetin on H_2_O_2_-induced cell cycle arrest, apoptosis and mitochondrial dysfunction was largely offset by ZnPP, an HO-1 inhibitor. This suggests that HO-1 activation was responsible for the blockade of H_2_O_2_-mediated oxidative damage by fisetin.

**Fig. 7:**
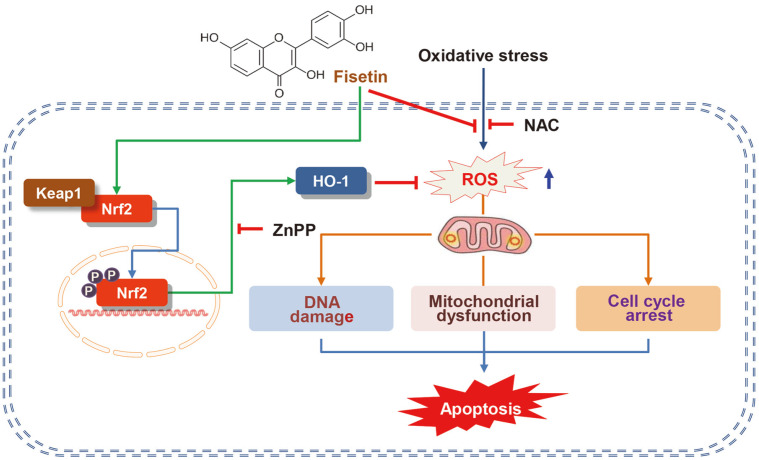
Schematic diagram of the blocking efficacy of fisetin on oxidative damage in C2C12 cells. As an activator of Nrf2 and a scavenger of ROS, fisetin protected cells from apoptosis by blocking H_2_O_2_-induced DNA and mitochondrial damage and cell cycle arrest.

In summary, our results indicated that fisetin can alleviate DNA damage, cell cycle perturbation and apoptotic cell death by mitigating H_2_O_2_-induced mitochondrial impairment and ROS generation in C2C12 myoblasts. In addition, fisetin, an activator of Nrf2, may contribute to the blockade of oxidative injury by activating of HO-1, indicating that fisetin has a high potential for application in the maintenance of myoblast function against oxidative damage (Fig. 7). However, additional studies are required to pinpoint the upstream signaling pathways controlling the activity of Nrf2 by fisetin and other intracellular pathways that may intervene in its antioxidant activity.
